# Telomere maintenance during anterior regeneration and aging in the freshwater annelid *Aeolosoma viride*

**DOI:** 10.1038/s41598-018-36396-y

**Published:** 2018-12-24

**Authors:** Chi-Fan Chen, Tzu-Ling Sung, Liuh-Yow Chen, Jiun-Hong Chen

**Affiliations:** 1 Department of Life Science, National Taiwan University, Taipei, Taiwan; 2 Institute of Molecular Biology, Academia Sinica, Taipei, Taiwan

## Abstract

Aging is a complex process involving declines in various cellular and physical functionalities, including regenerative ability. Telomere maintenance is thought to be necessary for regeneration, and telomere attrition is one mechanism that contributes to aging. However, it is unclear if aging affects regeneration owing to deterioration of telomeric maintenance. We introduce *Aeolosoma viride—*a freshwater annelid with strong regenerative abilities—as a new model for studying the effects of aging on telomere functions and regeneration. We show that the anterior regenerative ability of *A. viride* declines with age. We characterized the *A. viride* telomere sequence as being composed of TTAGGG repeats and identifyied the telomerase gene *Avi-tert*. In adult *A. viride*, telomerase was constantly active and telomere lengths were similar among different body sections and stably maintained with age. Notably, we found that regeneration did not result in telomere shortening at regenerating sites. Moreover, transient up-regulation of *Avi-tert* expression and telomerase activity was observed at regenerating sites, which might promote telomere lengthening to counteract telomere erosion resulting from cell proliferation. Our study suggests that although aging affects *A. viride* regeneration independent of steady-state telomere length, timely regulation of telomerase functions is critical for the regeneration process in *A. viride*.

## Introduction

Animals may repair or rebuild body parts through the regeneration process^1,2^. Regenerative abilities greatly differ among species and among the body parts of an individual^2–5^. Planarians have high regeneration capacity that can regenerate their entire body from a tiny fragment using pluripotent stem cells^4^. Salamanders can restore their appendages, but not their whole bodies, through de-differentiation of pre-existing cells and/or activation of resident progenitor cells^6^. Mammals are capable of rebuilding partially resected liver, but not brain^7^. Fetal mice can regenerate amputated digit tips, but this ability drastically decreases after birth^8^. As in mice, the regenerative abilities of most metazoans progressively decline with age^9–13^. For example, young killifishes can regenerate their caudal fin rapidly after amputation, but older killifishes take longer to do so and the outcomes can be flawed^14^. The regenerative abilities of mammals mainly rely on tissue-specific stem cells, which exhibit age-dependent functional declines^9,15^. Aging has been defined as “a progressive loss of physiological integrity, leading to impaired function and increased vulnerability to death”^16^. The underlying mechanisms of aging are multifaceted; both cell-intrinsic and -extrinsic mechanisms can be involved, one of which is telomere attrition^16–20^.

Telomeres are located at the ends of linear eukaryotic chromosomes and they are composed of telomeric DNA and associated proteins. Vertebrate telomeric DNA is composed of tandem TTAGGG repeats. The major function of telomeres is to protect chromosome ends from being recognized as DNA double-strand breaks and to prevent end-to-end fusions^21^. However, the inability to fully replicate the lagging strand causes the “end-replication problem” during DNA replication, which eventually leads to telomere shortening. When telomeres become too short to maintain genomic stability, the cell cycle is arrested by the DNA damage response mechanism, resulting in replicative senescence^22^.

Telomerase is a ribonucleoprotein (RNP) complex composed of an RNA component TERC and a reverse transcriptase TERT^23^. TERC assists in the assembly of telomerase and serves as a template for TERT to synthesize telomeric DNA. In unicellular organisms, telomerase is activated and required for indefinite proliferation^24^. However, in most multicellular organisms, telomerase activity is strictly regulated. In humans, telomerase is upregulated during embryonic development but, after birth, its expression is strongly restricted to germline cells and adult stem cells. Normal somatic tissues generally exhibit low or undetectable telomerase activity^22^, resulting in telomere shortening during cell replication. Although continuous erosion of telomeres limits the renewal capacity of cells and leads to age-related pathologies^16^, it is also considered as an intrinsic tumor suppression mechanism^25–27^.

Regeneration usually involves massive cell proliferation, during which telomere maintenance plays a significant role^3,4^. It has been proposed that telomere attrition results in the loss of self-renewal and proliferative abilities, which further impacts regeneration potential^9,15^. This idea is supported by studies on telomerase-deficient animals with severe regeneration-deficient phenotypes, characterized by stem cell depletion, impaired responses to tissue injury, and functional declines across multiple tissues and organs^28–32^. However, telomerase activity is upregulated in certain species with strong regenerative abilities, including several invertebrates, fish, amphibians, and reptiles^33–39^. Although there are established model animals for regeneration research, most of them have long lifespans and may be unsuitable for aging-related experiments^40^.

In this study, we introduced *Aeolosoma viride* (Annelida, Aphanoneura) as a new model for regeneration and aging-related research. *A. viride* is a freshwater annelid of 2 to 3 mm in length with 10 to 12 segments. *A. viride* exhibits profound regenerative capacity (this study & unpublished data). It can restore its lost anterior or posterior ends within a week. An entire animal can even be regenerated from a minimum of three body segments. *A. viride* reproduces asexually through paratomic fission at its posterior region, which often results in a linear chain of offspring. The lifespan of *A. viride* is approximately two months, making it a suitable organism for studying the influence of aging on regeneration. We also characterized the telomere sequence and telomerase of *A. viride* for the first time, and investigated changes in telomere length and telomerase activity during aging and regeneration. Our results indicate that *A. viride* is a feasible model species for studying the impact of aging on regeneration.

## Results

### Anterior regeneration of *A. viride* is sensitive to aging

**Figure 1:**
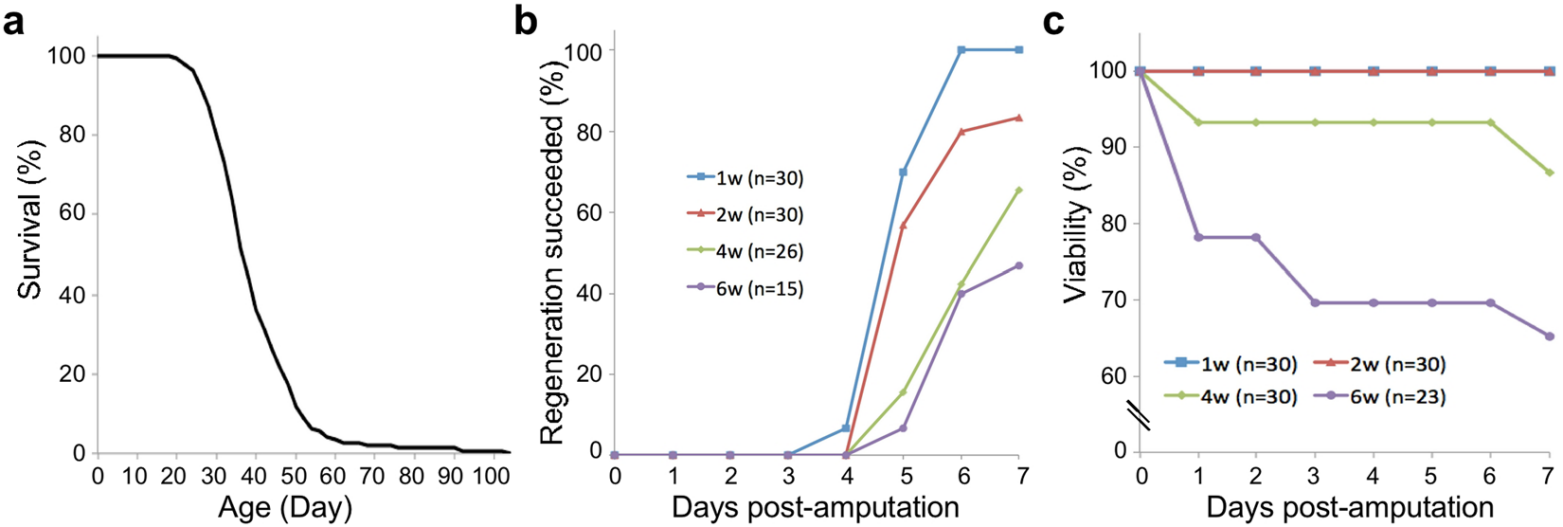
Effects of aging on anterior regeneration in *A. viride*. (**a**) Survival curve of *A. viride*. A total of 143 animals were cultured individually and observed every 48 hr. Mean survival time was 39.8 ± 11.8 days, with a maximum lifespan of 104 days and a minimum of 20 days. (**b**) Anterior regenerative ability of *A. viride* at different ages. 1-, 2-, 4-, and 6-week-old animals were synchronized by bisecting at the fission zone. After 3 days of recovery, the anterior segments were removed and percentages of animals that successfully regenerated were recorded. (**c**) Viabilities of *A. viride* during anterior regeneration at different ages.

To define the survival curve of *A. viride* under lab culture conditions, we isolated a total of 143 day-0 offspring and cultured them individually for observation. The lifespan of *A. viride* ranged from 20 to 104 days, with a mean survival time of 39.8 ± 11.8 days (Fig. 1a). Note that survivorship was high at early age, but decreased considerably by mid-lifespan. Thereafter, for the 10% of longest-living individuals, mean survival time increased to 63.6 ± 15.6 days.

Next, we investigated how aging influences head regeneration of the intact worms following anterior amputation. Although *A. viride* is able to regenerate different body parts, we chose to study head regeneration because in an intact worm the head region shows relatively low levels of cell proliferation compared to the tail region, which contains reproductive systems that could complicate our investigation of the regeneration process (unpublished data). To induce anterior regeneration, animals were bisected at the segment immediately anterior to the expanded midgut (Fig. S1). The head region was discarded and the body was kept to study anterior regeneration. Worms that restored their bulged head and could swim freely were considered successfully regenerated. Based on our lifespan analysis, we categorized *A. viride* into four age groups of 1-, 2-, 4-, and 6-weeks-old. All worms in the 1-week-old group (n = 30) and 80% of individuals in the 2-weeks-old group (n = 30) completed their anterior regeneration process within a week of observation (see Materials and Methods). In contrast, regeneration success decreased to 65% in the 4-weeks-old group, and it diminished further to 47% in the 6-weeks-old group (Fig. 1b). The 1- and 2-weeks-old age groups exhibited no mortality, but ~15% of animals in the 4-weeks-old group and ~35% in the 6-weeks-old group did not survive (Fig. 1c). These results indicate that the regenerative ability of the anterior region of *A. viride* declines with age.

### Identification of the telomeric DNA sequence of *A. viride*

**Figure 2:**
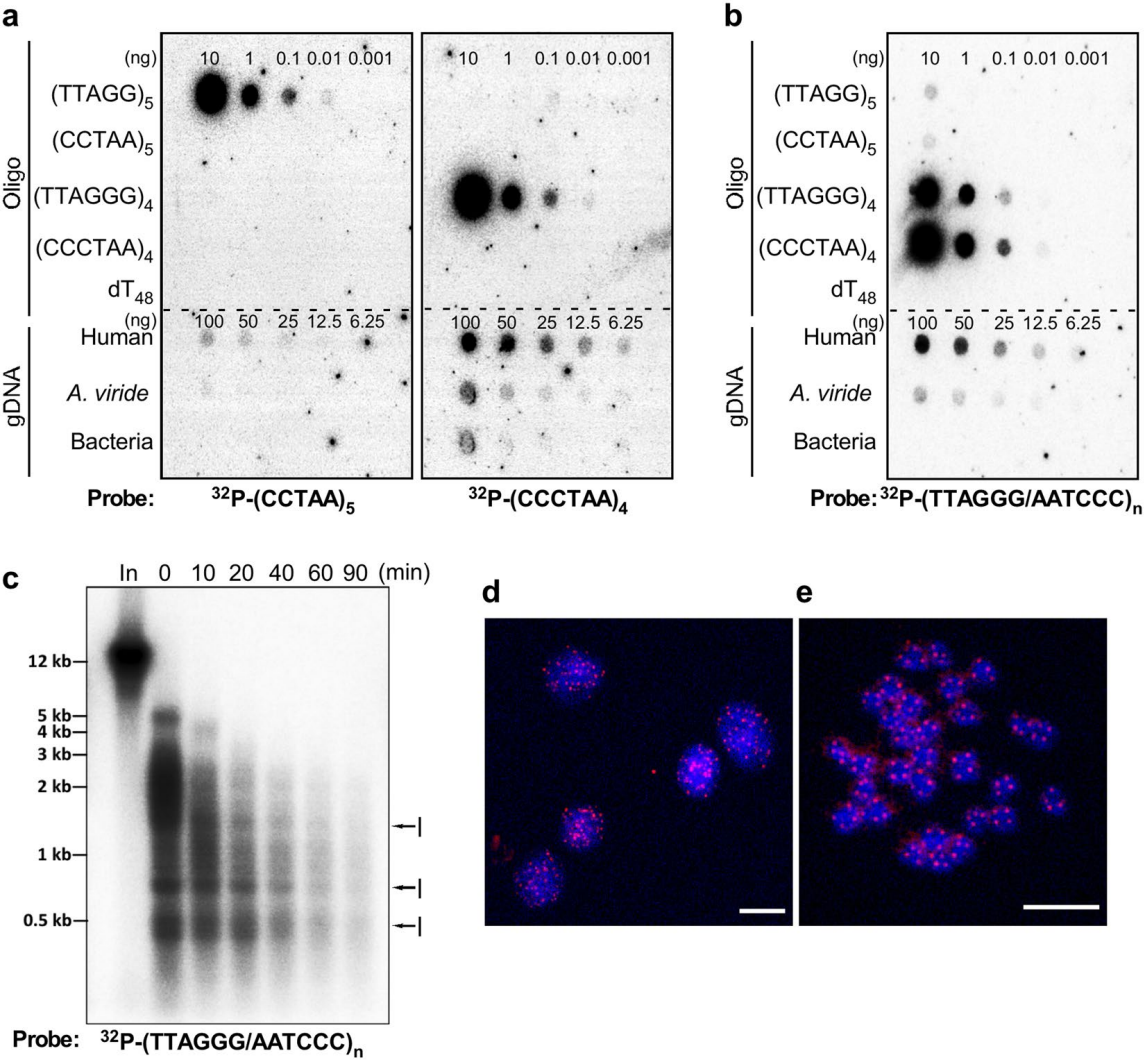
Identification of the telomeric DNA sequence in *A. viride*. Dot blotting of *A. viride* genomic DNA (gDNA) using oligonucleotide (**a**) or double-stranded (**b**) telomeric probes. Control oligonucleotides and genomic DNA were included for comparison. The oligo dT_48_ and bacterial genomic DNA are negative controls. (**c**) Telomeric sequences of *A. viride* are located at the ends of genomic DNA. Genomic DNA of *A. viride* was treated with Bal-31 exonuclease and then subjected to TRF assay using a double-stranded TTAGGG telomeric probe. In: intact genomic DNA. I: internal repetitive sequences. (**d,e**) *A. viride* telomeres detected by FISH in interphase nuclei (**d**) and metaphase spreads (**e**). Red fluorescent signals denote the TTAGGG telomeric sequences. Nuclei and chromosomes are in blue (DAPI). Scale bars: 10 μm.

In order to study *A. viride* telomere function and its correlation with aging and regeneration, we first endeavored to identify the *A. viride* telomere sequence. Two candidate telomere sequences, TTAGG and TTAGGG, were selected for examination based on a previous study by Traut *et al*.^41^. Accordingly, we used the corresponding oligonucleotide probes, (CCTAA)_5_ and (CCCTAA)_4_, in a dot blotting analysis. As shown in Fig. 2a, the TTAGGG repetitive sequence, rather than TTAGG, exists in *A. viride* genomic DNA (gDNA), as well as in human gDNA (lower panels). We confirmed probe specificity via a variety of dotted oligonucleotides, including those of the two candidate telomeric sequences and their complementary sequences (Fig. 2a, upper panels). The (CCCTAA)_4_ probe only detected the dotted (TTAGGG)_4_ oligo control, and the (CCTAA)_5_ probe only detected (TTAGG)_5_. We also used probes generated from double-stranded (TTAGGG)_n_ DNA and the data was consistent with the results from single-stranded oligos (Fig. 2b).

To reveal the chromosomal location of the repetitive TTAGGG sequence, we digested intact *A. viride* gDNA using Bal-31 exonuclease for various durations (up to 90 min) and then subjected it to a terminal restriction fragment (TRF) assay (Fig. 2c). Hybridization signals at 0 min revealed intact telomeres ranging from 1 to 5 kilobases (kb) in length, which gradually shortened within 10 minutes of exonuclease treatment and disappeared after 40 minutes of treatment. The sensitivity to exonuclease digestion confirms that *A. viride* telomeric sequences are terminal. Note that some interstitial TTAGGG sequences also exist (denoted as I in Fig. 2c), which were insensitive to exonuclease digestion. Furthermore, we performed molecular cytogenetic analysis of *A. viride* by telomere fluorescence *in situ* hybridization (telomere FISH). Using a (CCCTAA)_3_ peptide nucleic acid (PNA) probe, we observed that telomeric signals were randomly scattered within interphase nuclei (Fig. 2d) and localized at chromatid ends in metaphase spreads (Fig. 2e). Thus, we conclude that the *A. viride* telomeric sequence is “TTAGGG” and that it is located at chromosome ends.

### Telomere length is maintained during anterior regeneration in *A. viride*

**Figure 3:**
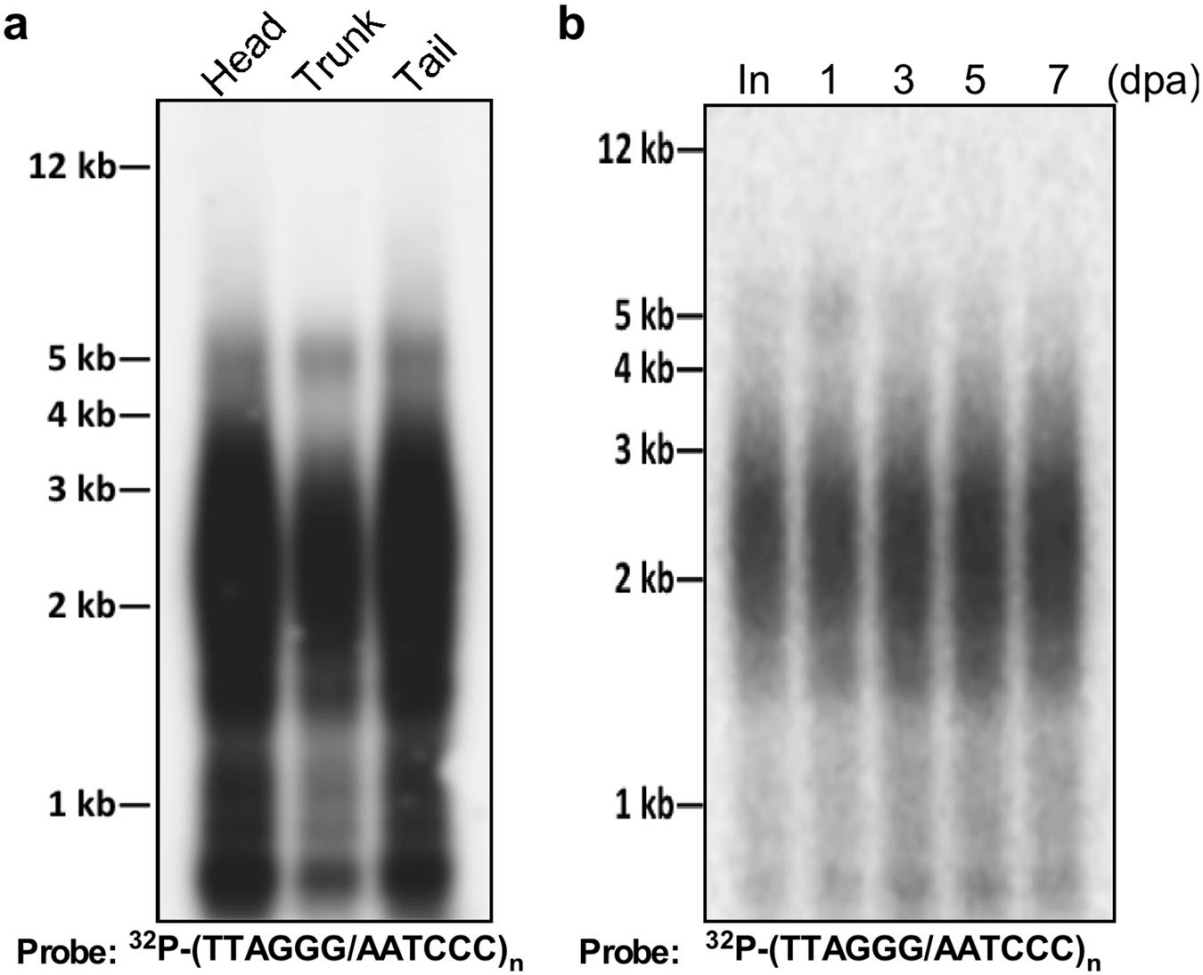
Telomere length maintenance in intact or regenerating *A. viride*. TRF assay was used to measure the telomere lengths of *A. viride* in different body parts (**a**), including the head, trunk, and tail (see Fig. 6a for illustration), or regenerating sites from 1 to 7 days post-amputation (dpa) (**b**). The blot was probed with a double-stranded TTAGGG telomeric probe.

We investigated if *A. viride* telomere length varies in different body sections or during regeneration. As shown in Fig. 3a, the telomere lengths of *A. viride* in the head, trunk, and tail sections (see Fig. 6a for illustration) were nearly identical. Following head amputation, telomere length at the regenerating site was maintained over 7 days post-amputation (dpa) and it was comparable to that of intact animals (Fig. 3b). This outcome suggests that *A. viride* possesses a telomere elongation mechanism to counteract the telomere erosion associated with DNA replication during rapid cell proliferation for anterior regeneration.

**Figure 4:**
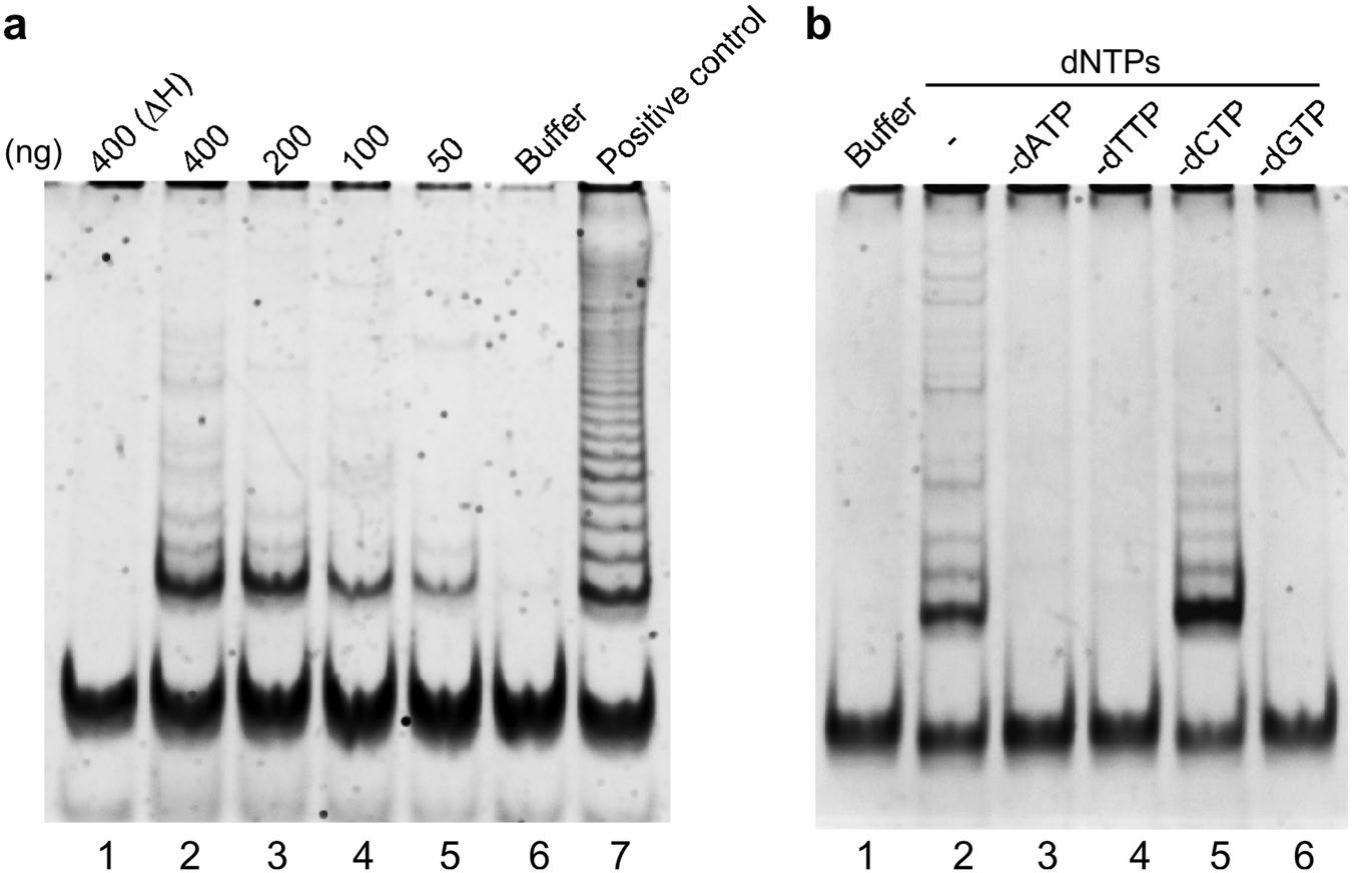
Detection of telomerase activity by TRAP assay in *A. viride*. (**a**) Telomerase activity in *A. viride*. The amount of protein extract used in TRAP reaction is indicated above each lane. A ladder-like pattern indicates telomerase-extended products amplified by PCR reactions, and the fast-running band in each lane represents the 36 bp internal control. ΔH: heat-inactivated control. Buffer: lysis buffer. Positive control: lysate from telomerase-overexpressed 293T human cells. (**b**) Deoxyribonucleoside triphosphates (dNTPs) required for telomerase reaction. TRAP reactions were performed with dNTPs (−) or with exclusion of individual dNTPs (-dATP, -dTTP, -dCTP, and -dGTP). Subsequent PCR reaction was performed with dNTPs.

**Figure 5:**
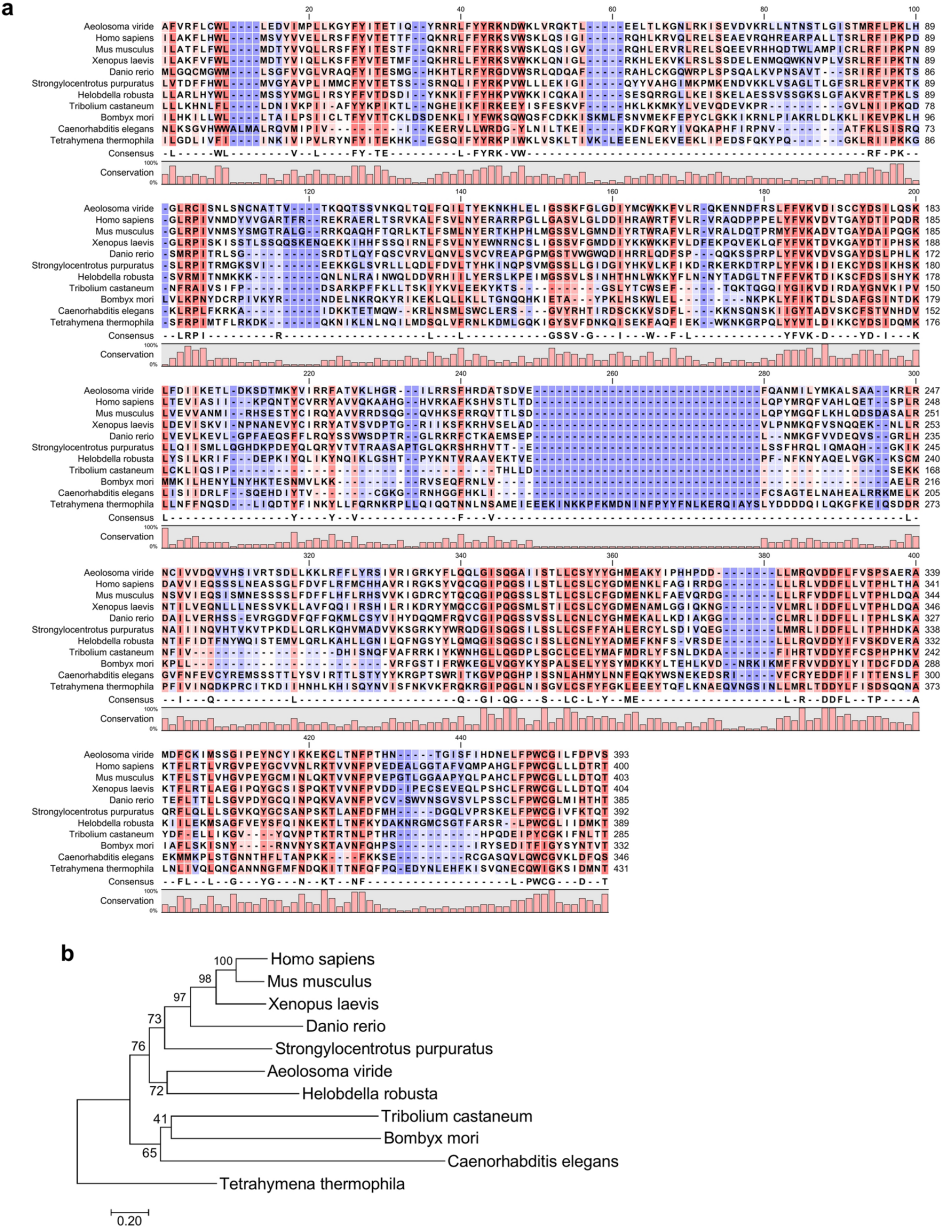
Multiple sequence alignment and phylogenetic analysis of *Avi*-TERT. (**a**) Multiple sequence alignment of the conserved telomerase-specific motif T from the TRBD domain and the entire RT domain of TERT from 11 representative species, including human (*Homo sapiens*), mouse (*Mus* musculus), clawed frog (*Xenopus laevis*), zebrafish (*Danio rerio*), sea urchin (*Strongylocentrotus purpuratus*), leech (*Helobdella robusta*), insects (*Tribolium castaneum* and *Bombyx mori*), nematode (*Caenorhabditis elegans*), and a ciliate (*Tetrahymena thermophila*). Consensus residues (>60% identity) are shown in the bottom row of the alignment. Conservation of each aligned residue is shown below each panel as bar plots. (**b**) Phylogenetic tree of TERTs from 11 representative species, constructed using the neighbor-joining method. The number next to each node indicates nodal support as a percentage of 2000 bootstrap replicates.

**Figure 6:**
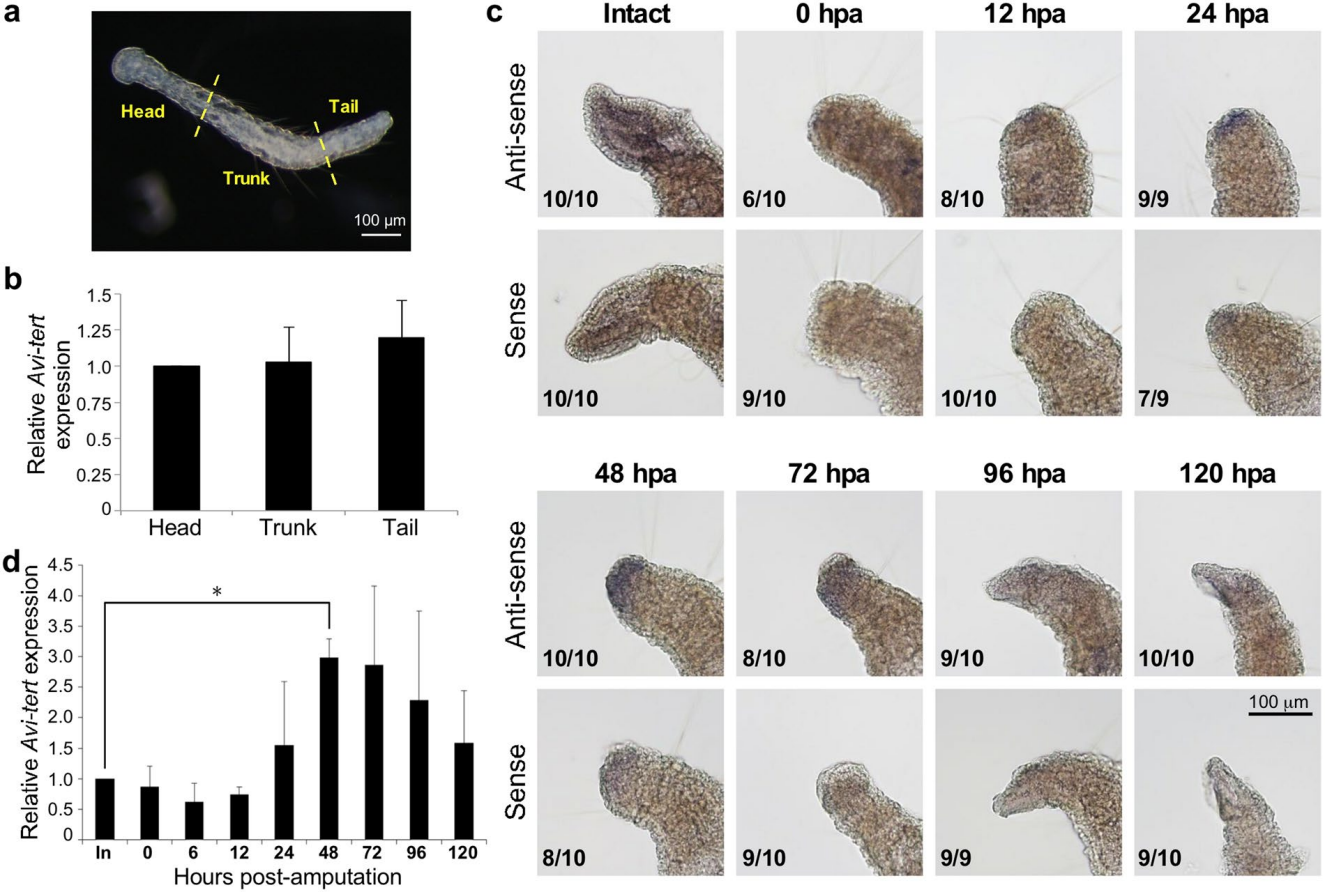
Expression of *Avi-tert* in intact or anterior regenerating *A. viride*. (**a**) Tissue collection from *A. viride*. Individual animals were bisected into three parts, head (including mouth and pharynx), trunk (entire midgut), and tail (including the fission zone and anus) for subsequent analyses. Scale bar: 100 μm (**b**) *Avi-tert* mRNA expression levels in different body parts of *A. viride*. The expression level was first normalized to *Avi-β-actin* mRNA and then to the normalized value of the head region. Error bars represent standard deviation (n = 3 biological replicates). (**c**) WISH analyses revealing *Avi-tert* expression during anterior regeneration. An anti-sense probe was used for *Avi-tert* detection and a sense probe acted as a negative control. Fractions at the lower left side of each picture indicate the numbers of animals exhibiting the respective expression pattern out of the total number of examined individuals. (**d**) Quantification of the level of *Avi-tert* mRNA during anterior regeneration in *A. viride*. The expression level was first normalized to *Avi-β-actin* and then to the normalized value of the intact head. Error bars represent standard deviation (n = 3 biological replicates). *p < 0.05.

**Figure 7:**
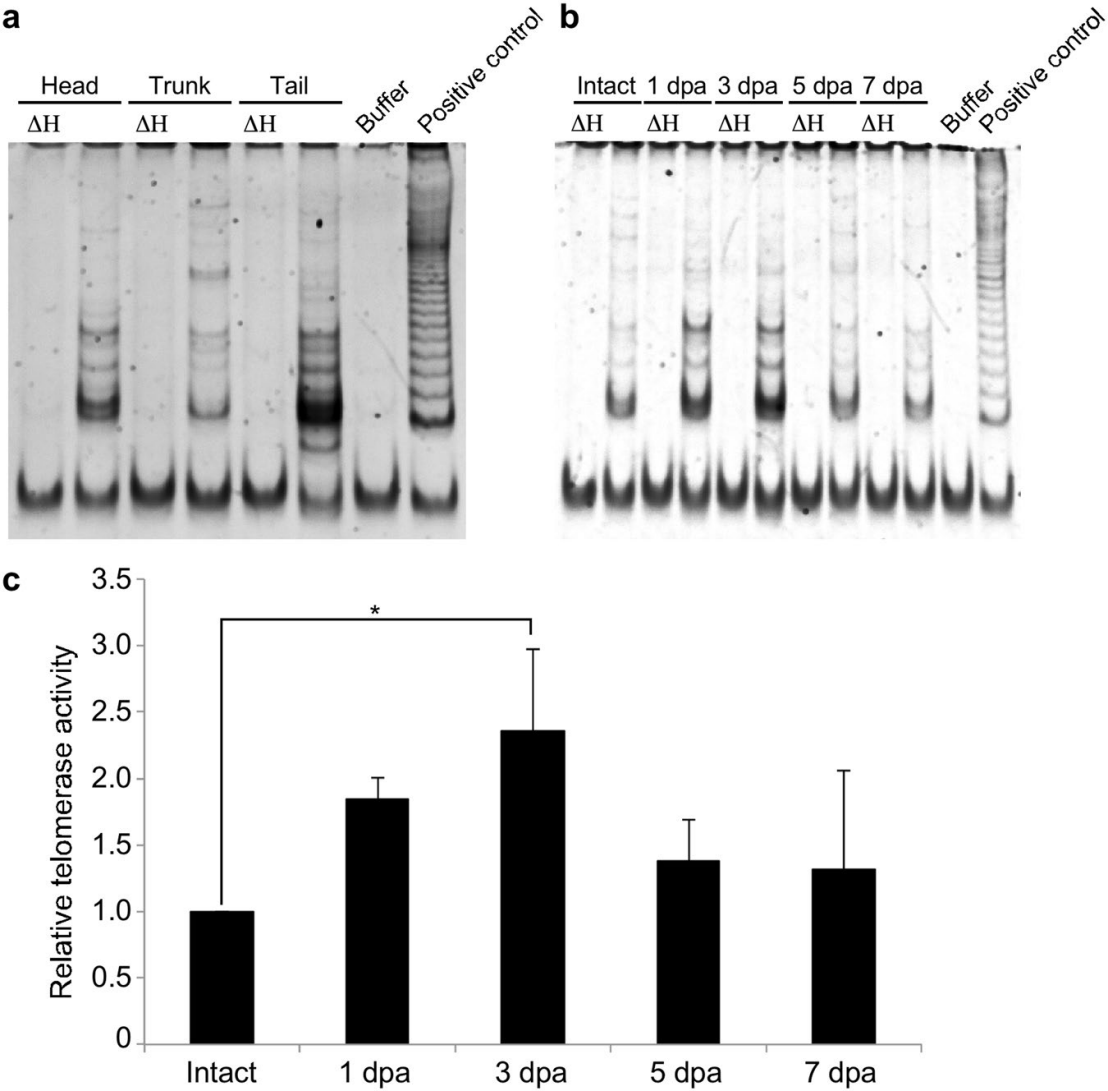
Telomerase activity in intact or anterior regenerating *A. viride*. TRAP assays were used to measure the telomerase activity of *A. viride* in different body parts, including head, trunk, and tail (**a**), or regenerating sites from 1 to 7 days post-amputation (dpa) (**b**). A ladder-like pattern indicates telomerase-extended products amplified by PCR reactions, and the fast-running band in each lane represents the 36 bp internal control. ΔH: heat-inactivated control. Buffer: lysis buffer. Positive control: lysate from telomerase-overexpressed 293T human cells. (**c**) Quantification of telomerase activity detected by TRAP assay during anterior regeneration of *A. viride*. Activities were first normalized to the corresponding internal control bands and then to the normalized activity of intact animals (relative telomerase activity = 1). Error bars represent standard deviation (n = 3 biological replicates). *p < 0.05.

**Figure 8:**
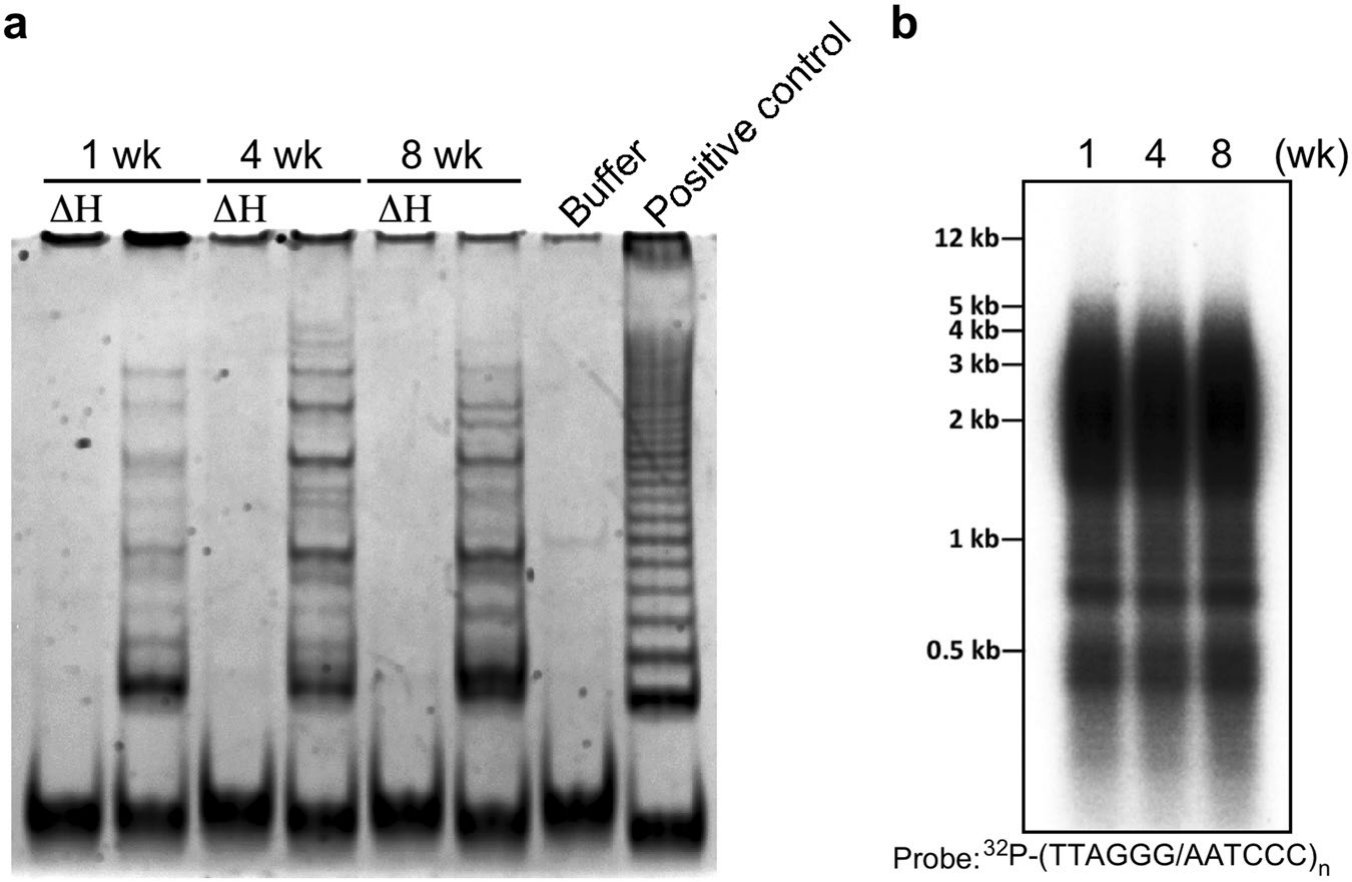
Telomerase activity and telomere length maintenance during *A. viride* aging. (**a**) Telomerase activity was detectable in 1-, 4-, and 8-week-old animals. A ladder-like pattern indicates telomerase-extended products amplified by PCR reactions, and the fast-running band in each lane represents the 36 bp internal control. ΔH: heat-inactivated control. Buffer: lysis buffer. Positive control: lysate from telomerase-overexpressed 293T human cells. (**b**) TRF assay was performed to measure telomere lengths in the three age group.

### The Presence of telomerase in *A. viride*

We then used a telomeric repeat amplification protocol (TRAP) assay to detect telomerase activity in *A. viride*. Whole-animal protein extract generated a ladder-like pattern (Fig. 4a, lanes 2–5), which is considered a typical output resulting from telomerase activity. This activity is sensitive to protein denaturation, since heat inactivation completely abolished the effect (Fig. 4a, lane 1). If we excluded dATP, dTTP, dCTP, or dGTP at the elongation step, only in the absence of dCTP was telomerase activity not affected (Fig. 4b, lane 5). This result is consistent with the fact that dCTP is not required to elongate the telomeric sequence of TTAGGG and confirms the existence of a telomerase in *A. viride*.

### Molecular cloning of an *A. viride* telomerase reverse transcriptase gene

From the *A. viride* transcriptome database, we found an annotated partial sequence of a putative telomerase gene *tert*. Using 5′ and 3′ rapid amplification of cDNA ends (RACE), we could extend the partial sequence and constructed a *tert* cDNA of 5389 base pairs (bp) in length, which comprised a 3600-bp open reading frame (ORF), a 143-bp 5′ untranslated region (5′ UTR), and a 1646-bp 3′ untranslated region (3′ UTR) (Fig. S2). The ORF encodes a polypeptide of 1199 amino acids (Fig. S3), with a calculated molecular weight of 137.27 kDa and an isoelectric point of 9.44. Using the NCBI BLASTp search tool, we identified two specific domain hits from the translated ORF: (1) a telomerase RNA-binding domain (TRBD) from residue 551 to 669, and (2) a reverse transcriptase (RT) domain from residue 882 to 1012. Through alignment with human TERT, N-terminal residues 1 to 187 and C-terminal residues 1016 to 1199 were annotated as the telomerase essential N-terminal (TEN) domain and the C-terminal extension (CTE) domain, respectively (Fig. S4). Hereafter, we refer to the identified gene as *Avi-tert* and the telomerase protein as *Avi-*TERT.

To compare *Avi-*TERT with TERTs from other species, we performed a global alignment incorporating the TERT polypeptide sequences of *A. viride*, *Homo sapiens*, *Mus musculus*, *Xenopus laevis*, *Danio rerio*, *Strongylocentrotus purpuratus*, *Helobdella robusta*, *Tribolium castaneum*, *Bombyx mori*, *Caenorhabditis elegans*, and *Tetrahymena thermophila*. Our results reveal that *Avi*-TERT shares 27% sequence identity with TERTs from *H. sapiens*, *M. musculus*, *X. laevis* and *H. robusta*, and 25% identity with those from *D. rerio* and *S. purpuratus*. *Avi*-TERT shared less than 20% identity with TERTs from *T. castaneum*, *B. mori*, *C. elegans*, and *T. thermophila*. Alignments of telomerase-specific motif T and the RT domain are shown in Fig. 5a. We constructed a phylogenetic tree based on this alignment, which grouped all vertebrate TERTs tightly in a subgroup, whereas the invertebrate TERTs were more diversely branched (Fig. 5b). The phylogenetic analysis revealed that *Avi*-TERT was closely related to a homolog from another annelid *H. robusta*. The TERT of *S. purpuratus* was clustered with four vertebrate TERTs in a deuterostome clade. The TERTs of the nematode (*C. elegans*) and two insects (*T. castaneum* and *B. mori*) formed an ecdysozoan clade. The TERT of *T. thermophila*, a ciliate protozoan, served as an outgroup. Thus, our overall TERT phylogeny is consistent with currently accepted evolutionary relationships.

### Telomerase expression and activity are upregulated during *A. viride* anterior regeneration

Expression of *Avi-tert*, measured by RT real-time PCR, was comparable in different sections of *A. viride* (Fig. 6a,b). To determine if expression of *Avi-tert* fluctuates during anterior regeneration, we conducted whole-mount *in situ* hybridization (WISH) to visualize *Avi-tert* mRNA in regenerating animals. As shown in Fig. 6c, *Avi-tert* mRNA was first detected by the anti-sense probe at 24 hours post-amputation (hpa), peaked at 48 hpa, and disappeared at 120 hpa. We also evaluated *Avi-tert* mRNA levels at the regenerating site by RT real-time PCR (Fig. 6d). Consistent with our WISH data, relative expression of *Avi-tert* was upregulated at 24 hpa, had increased three-fold at 48 hpa, and then steadily decreased thereafter.

We then examined telomerase activity by TRAP assay. In intact animals, telomerase activity was detected in head, trunk, and tail sections, with the tail showing the strongest and the trunk the lowest activities (Fig. 7a). However, as already stated, telomere lengths in these three body sections were relatively similar (Fig. 3a), suggesting the presence of a length control mechanism. We collected tissue from regenerating sites and found that telomerase activity was upregulated from 1 to 3 dpa and then returned to near basal level at 5 dpa (Fig. 7b,c). This up-regulation of telomerase activity is likely required to maintain telomere length during regeneration (Fig. 3b). These results indicate that *Avi-tert* expression and *Avi-*TERT activity are upregulated at the regenerating site after amputation.

### Telomere length is maintained as *A. viride* age

To investigate telomere maintenance during the aging process of *A. viride*, we examined telomerase activity and telomere length of 1-, 4-, and 8-week-old animals. Telomerase activity was detectable throughout the lifespan of *A. viride* but was higher in 4- and 8-week-old animals (Fig. 8a). Telomere length was similar between all three age groups (Fig. 8b). These results indicate that *A. viride* telomere length does not shorten with aging, presumably maintained by constitutively active telomerase.

## Discussion

In this study, we identified TTAGGG as the telomeric sequence of *A. viride*, which is the ancestral telomeric motif identified in basal metazoans such as sponges and cnidarians^41^. This sequence is present in diverse metazoan species including chordates, echinoderms, mollusks, and platyhelminthes, but has been lost in ecdysozoans such as nematodes and arthropods^42^. Importantly, in addition to *A. viride*, other members of Annelida—including marine worms, earthworms, and leeches—possess the same telomeric sequence^43–45^. Our phylogenetic analysis clustered *Avi*-TERT with a homologous protein from another annelid, with both these proteins exhibiting closer evolutionary relationships with the homologs of deuterostomes than of ecdysozoans (Fig. 5). Although annelid and ecdysozoan species are protostomes, divergent telomeric DNA and TERT protein sequences may represent evolutionary differences in their telomere biology.

We detected constitutive *Avi-tert* gene expression as well as telomerase activity in different body sections (Figs 4,6b and 7a), which have also been observed in some aquatic animals with high regenerative abilities^36,46–49^. In red sea urchin (*Mesocentrotus franciscanus*), telomerase activity has been detected in various adult tissues, including Aristotle’s lantern muscle, esophagus, intestine, ampullae, and gonad^50^. It is conceivable that the constant active telomerase activity may be critical to support tissue homeostasis and strong regeneration ability. Interestingly, we found strong telomerase activity in the tail section compared to the head and trunk regions of *A. viride* (Fig. 7a). The difference in telomerase activity is not due to *Avi-tert* mRNA expression, suggesting that telomerase function may be post-transcriptionally regulated in the tail region. We note that the reproduction zone is located in the tail region^51^, where high cell proliferation may result in shortened telomeres. However, given our observation of similar telomere lengths in all body sections, the elevated telomerase activity in the tail region is likely important to maintain length homeostasis of telomeres in this active growth zone. In fact, this strategy is also evident in the asexual strain, but not sexual strain, of planarian that possesses high levels of telomerase activity for telomere length maintenance during reproduction by fission^33^.

We also found evidence for upregulation of *Avi-tert* expression and telomerase activity during anterior regeneration, consistent with what has been observed for other animals with strong regenerative abilities^33–39^. Interestingly, the length of *A. viride* telomeres is also maintained during the regenerative process, indicating that some control mechanisms may exist to ensure telomere length homeostasis. Two essential telomere-associated protein complexes, shelterin and CST (CTC1-STN1-TEN1), have been shown to be involved in telomere homeostasis in mammals^52,53^. Shelterin interacts with telomeric DNA to protect telomeres from being recognized by the DNA repair machinery, and it also recruits telomerase to the telomere ends during telomere replication. CST, a telomere end-binding complex, specifically binds to newly synthesized telomeric 3′ overhangs and terminates telomerase-mediated telomere elongation. In an attempt to search for shelterin and CST components in *A. viride* using a transcriptome database, we identified homologs of *pot1* (a shelterin component) and *ten1* (a CST component). It would be interesting to investigate the functions of these homologs in *A*. *viride*.

Telomere attrition is one of the factors involved in the aging process. However, we did not uncover evidence of telomere shortening during the lifespan of *A. viride* in this study and, in fact, we found that telomerase activity is sustained in aged animals. These results indicate that telomere length shortening might not be the primary factor involved in *A. viride* aging. One possible explanation is that the lifespan of *A. viride* is relatively short to be affected by telomere attrition. A previous study on *Daphnia* showed that only the long-lived *D. pulicaria* demonstrates an age-associated decline in telomerase activity and telomere length, whereas the short-lived *D. pulex* maintains its telomerase activity and telomere length throughout its lifespan^54^. In addition, a study by Canistro *et al*. suggests that oxidative stress is a critical factor controlling aging in *A. viride*^55^. Although our results might indicate that telomere-related dysfunction is less influential on aging, it will be necessary to employ inhibitory methods, such as *Avi-tert* gene silencing or drugs that inhibit telomerase activity, to examine if *A. viride* telomerase activity contributes directly to telomere length maintenance.

Aging is a consequence of multiple molecular mechanisms that together influence the lifespan of an organism. In this study, we have identified the telomere sequence and telomerase of *A. viride*, and demonstrated that telomerase is active throughout this animal’s lifespan. Although the anterior regenerative ability of *A. viride* declines with age, telomere length is maintained, suggesting the involvement of other factors in the aging process. Further investigations are required to understand the aging process of *A. viride* and the underlying mechanisms leading to regeneration failure in older animals.

## Methods

### Animal cultures and anterior regeneration procedure

*A. viride* were cultured in artificial spring water (ASW, 48 mg/L NaHCO_3_, 24 mg/L CaSO_4_·2H_2_O, 30 mg/L MgSO_4_·7H_2_O, and 2 mg/L KCl in ddH_2_O) at 23 ± 1 °C and under a photoperiod of 12 hours light and 12 hours darkness. Animals were fed with powdered oats twice a week. Several pools of animals were maintained for experiments.

To generate even-aged cohorts, a number of animals were isolated and cultured individually. The next day, offspring from the isolated animals were collected and maintained individually in 24-well plates, each with 500 μL ASW. Each worm was fed with 10 mg (dry weight) powdered oats and observed every 48 hours. The culture medium was changed every six days.

Prior to anterior end amputation, *A. viride* were kept in tap water for 10 minutes and then starved in ASW overnight. To minimize the influence of reproduction between animals in different fission stages, animals were bisected at the segment anterior to the fission zone for synchronization and kept at 25 °C. After 3 days of recovery, the anterior segments comprising the entire head were amputated, and the animals were transferred into fresh ASW for subsequent experiments (Fig. S1).

### DNA, RNA, and protein preparations

To obtain gDNA from different age groups, 80 synchronized individuals were pooled. For regenerating tissues, the most anterior trunk segments from 200 amputated individuals were pooled. Samples were maintained in Nuclei Lysis Solution (Promega) at −20 °C. Extractions were done using the Wizard^®^ Genomic DNA Purification Kit (Promega) following the manufacturer’s instructions. Quality and concentration of the isolated DNA was determined using a NanoDrop ND-1000 (Thermo Scientific), and DNA integrity was checked in a 1% agarose gel.

For total RNA extraction, regenerating tissues from 55 individuals were pooled and then stored in Trizol reagent (Invitrogen) at −20 °C until extraction. Reverse transcription was performed with the SuperScript^®^ III First-Strand Synthesis System for RT-PCR (Invitrogen) using either oligo dT or random hexamers.

To prepare protein extracts, 30 individuals, whose asexual reproduction zone had been removed, were pooled. We used 100 individuals when protein extracts exclusively from regenerating tissues were needed. Samples were suspended in ice-cold CHAPS Lysis Buffer (Millipore), homogenized by pipetting, and then incubated on ice for 30 minutes. After centrifugation at 12000 × *g* for 20 minutes at 4 °C, the supernatant was maintained as a protein extract and stored at −80 °C. Concentration was determined by Bradford Reagent (Sigma-Aldrich).

### Dot blotting

Single-stranded oligonucleotides and gDNA were mixed with an equal volume of 0.4 M NaOH and then incubated at 95 °C for 5 minutes for denaturation. After neutralizing with 2 M NH_4_OAc, samples were dotted onto membrane using a Bio-Dot^®^ Microfiltration Apparatus (Bio-Rad) according to the manufacturer’s instructions.

For probe hybridization, membranes were blocked with pre-warmed Church buffer (0.5 M NaPO_4_, 1 mM EDTA, 7% SDS, and 1% BSA in nuclease-free water) and incubated at 45 °C for 1 hour. Oligonucleotide probes were then added for hybridization at 45 °C overnight. After washing, membranes were exposed to a phosphor screen overnight and then scanned with a Typhoon^TM^ FLA9000 biomolecular imager (GE Healthcare). Hybridization was performed at 50 °C when the double-stranded TTAGGG probe was used.

The oligonucleotide probes (CCTAA)_5_ and (CCCTAA)_4_ were labeled with ^32^P by T4 polynucleotide kinase (NEB) using [γ-^32^P]ATP according to the manufacturer’s instructions. For double-stranded telomeric probes, double-stranded (TTAGGG)_n_ DNA was first generated by a self-priming PCR reaction using (TTAGGG)_4_ and (CCCTAA)_4_ primers. Subsequently, ^32^P-labled probes were generated using the RadPrime^TM^ DNA Labeling System (Invitrogen) with [α-^32^P]dCTP. After labeling, probes were purified with illustra^TM^ MicroSpin^TM^ G-25 Columns (GE Healthcare). Before hybridization, probes were heated for 10 minutes, chilled on ice, and then added to the membrane.

### Bal-31 exonuclease digestion

Exonuclease digestion was performed at 30 °C using Bal-31 (NEB). Aliquots of the reaction were terminated with EGTA (final concentration of 20 mM) followed by 65 °C incubation for 10 mins. After phenol-chloroform extraction, the digested DNA was precipitated with GlycoBlue^TM^ coprecipitant (Ambion) in isopropanol to enhance yield.

### Terminal restriction fragment (TRF) assay

Genomic DNA was digested with an *Rsa*I and *Hinf*I (NEB) endonuclease mixture (1:1) at 37 °C overnight and then resolved in a 1% agarose gel. After electrophoresis, the gel was stained with FluoroStain™ DNA Fluorescent Staining Dye (SMOBIO) to check the quality of DNA digestion. The gel was then soaked in 0.25 N HCl for 15 minutes to depurinate the DNA. After briefly rinsing with distilled water, the gel was gently rocked twice in denaturing solution (0.5 N NaOH, 1.5 M NaCl) for 15 minutes, rinsed again, and then neutralized twice with neutralizing solution (1.5 M NaCl, 1M Tris-HCl, pH 7.5) for 15 minutes. The DNA fragments were then blotted onto a nylon membrane (Immobilon™-Ny^+^; Millipore) by capillary transfer in 10x SSC overnight. The blotted membrane was briefly rinsed with 10x SSC, air-dried, and then crosslinked by exposing it to 120 mJ/cm^2^ of UV light. Southern hybridization was carried out as described above.

### Telomere fluorescence *in situ* hybridization (Telomere FISH)

Regenerating tails were fixed in ice-cold Carnoy’s fixative (methanol/glacial acetic acid, 3:1) for telomere FISH. Fixed tissues were dissociated in 60% acetic acid with needles on a coverslip, heated at 70 °C for 1 minute, and then air-dried overnight. Before processing, samples were rehydrated in PBS and fixed with 4% PFA/PBS for 10 minutes. After PBS washes, hybridization was performed using 250 μM (CCCTAA)_3_ peptide nucleic acid (PNA) probe (Panagene) in hybridization mix (70% formamide, 0.5% Blocking reagent (Roche), 10 mM Tris-HCl, pH 7.5). After post-hybridization washes, samples were counterstained with 0.3 μM DAPI, dehydrated in an ethanol series (70%, 95% and 100%, each for 5 minutes), and then mounted in ProLong^®^ Gold Antifade Mountant (Molecular Probes).

### Telomeric repeat amplification protocol (TRAP) assay

TRAP assays were performed using a TRAPeze Telomerase Detection kit (Millipore), following the manufacturer’s protocol. Each TRAP reaction contained 200 ng (unless specified otherwise) of *A. viride* protein extract in a 25 μL reaction volume. Reaction products were mixed with FluoroDye™ DNA Fluorescent Loading Dye (SMOBIO), resolved in a 10% non-denaturing polyacrylamide gel, and visualized using the FluorChem M system (ProteinSimple). We analyzed intensities of TRAP products using ImageJ (NIH), and an internal control band was used to normalize and quantify relative telomerase activities. Statistical analysis was conducted using one-way analysis of variance (ANOVA) followed by Dunnett’s post hoc test.

### Gene cloning and sequence analysis

A partial sequence of *Avi-tert* was identified from analysis of the *A. viride* transcriptome. Gene-specific primers were used to amplify the partial cDNA fragment of *Avi-tert* using Supertherm Tag DNA polymerase (Bersing). The PCR product was purified and the DNA was cloned using a T&A^TM^ Cloning Kit (Yeastern Biotech) for sequencing.

To extend the partial sequence of *Avi-tert*, we performed 5′ and 3′ rapid amplification of cDNA ends (5′ and 3′RACE). For 5′RACE, a 5′ adaptor primer (5′ AP: 5′-GGCCACGCGTCGACTAGTACGGGGGGGGGGGGGGGG-3′) and an adaptor primer (AP: 5′-GGCCACGCGTCGACTAGTAC-3′) were used. For 3′RACE, two gene-specific forward primers, RACE-1: 5′-GTATTAGGTCACGTGTTGTTGCCAACA-3′ and RACE-2: 5′-CAGGCGTGCACCACAACTTATACAATC-3′, near the 3′ end of the partial sequence were used. We used cDNA synthesized with oligo (dT) primers as templates for the first-round PCR with RACE-1 primer and a 3′ adaptor primer (3′ AP: 5′-GGCCACGCGTCGACTAGTACTTTTTTTTTTTTTTTTTTT-3′). The second-round PCR was then carried out with RACE-2 primer and AP. The final PCR products from 5′ and 3′RACE were cloned and sequenced to obtain partial sequences of *Avi-tert*, which were then further extended. Homology analysis was performed by BLASTx or BLASTp in the NCBI website_._

Multiple sequence alignment was performed using the MUSCLE program with default parameters in MEGA 7.0. TERT sequences used were from *Homo sapiens* (NP_937983.2), *Mus musculus* (AAC09323.1), *Xenopus laevis* (AAG43537.1), *Danio rerio* (ABM92944.1), *Strongylocentrotus purpuratus* (NP_001165522.1), *Helobdella robusta* (DAA35191.1), *Tribolium castaneum* (NP_001035796.1), *Bombyx mori* (ABF56516.1), *Caenorhabditis elegans* (NP_492373.1), and *Tetrahymena thermophila* (AAC39135.1). The alignment was exported and then graphed using CLC Sequence Viewer 6 (CLC bio A/S). We constructed the phylogenetic tree using the neighbor-joining method in MEGA 7.0. Tree node reliability was assessed in MEGA 7.0 using 2000 bootstrap replicates.

### Real-time PCR analysis

Real-time PCR was performed using IQ™ SYBR^®^ Green Supermix (Bio-Rad) and a CFX96 Touch™ Real-Time PCR Detection System (Bio-Rad). For each sample, the gene expression level was normalized using the internal control gene *β-actin*. Statistical analysis was done by one-way ANOVA followed by Dunnett’s post hoc test. The primers were: *Avi-tert* qPCR primers (forward: 5′-GCAAGTAGCCAGCGAAAGAG-3′, reverse: 5′-GCACCCACACCTCCATTATTAA-3′); *Avi-β-actin* qPCR primers (forward: 5′-GGAGATCTCTGCTCTTGCCC-3′, reverse: 5′-GGAGTACTTG CGCTCAGGTG-3′).

### Whole-mount *in situ* hybridization (WISH)

Animals were fixed in 4% PFA/PBS at 4 °C overnight and then washed five times with PBST (PBS containing 0.1% Tween-20) for 5 minutes and dehydrated in methanol at −20 °C overnight. Before hybridization, dehydrated samples were re-hydrated and then treated with proteinase K (10 μg/mL) for 10 minutes. After post-fixation in 4% PFA for 20 minutes, pre-hybridization was carried out with HYB buffer (50% formamide, 5x SSC, 50 μg/mL heparin, 500 μg/mL torula RNA type VI (Sigma-Aldrich), 9.2 mM citric acid, 1x Denhardt’s Solution, and 0.1% Tween-20 in nuclease-free water) at 58 °C overnight.

Samples were hybridized with riboprobes (1 ng/μL) at 58 °C for 24 hours. After HYB buffer washing and gradient transfers (66%, 33%, and 0% in 2x SSCT at 58 °C), we performed two stringent washes (0.2x SSCT at 58 °C for 15 minutes) followed by a series of 0.2x SSCT gradient transfers (66%, 33%, and 0% in PBST at 25 °C). Samples were then blocked with 5% bovine serum albumin before being incubated with anti-DIG-AP Fab fragments (Roche) at 4 °C overnight. After washing, samples were stained with NBT (400 μg/mL) and BCIP (200 μg/mL) at 25 °C in the dark and then mounted in glycerol.

To prepare riboprobes for WISH, the target sequence was amplified by PCR using specific primers (forward: 5′-CCATGTGCCTGTAATGGTTGCA-3′, reverse: 5′-CTACCACCTGAGAATCCTTCATG-3′) and cloned using a T&A^TM^ Cloning Kit (Yeastern Biotech). The sense or anti-sense riboprobes were labeled with digoxigenin (DIG) through incorporation of DIG-11-UTP during *in vitro* transcription. Finally, the DIG-labeled riboprobes were purified and dissolved in HYB buffer and then stored at −20 °C.

## Electronic supplementary material

Supplementary Figures

Dataset 1

## Data Availability

The datasets generated or analyzed during this study are included in this published article (and its Supplementary information).
