# Supplementary material for: Telomere maintenance during anterior regeneration and aging in the freshwater annelid Aeolosoma viride
Source: Sci Rep. 2018 Dec 24;8:18078. doi: 10.1038/s41598-018-36396-y (PMC6305377; doi:10.1038/s41598-018-36396-y)
Supplement: Supplementary file 1 — Supplementary Figures [file 41598_2018_36396_MOESM1_ESM.pdf]

Supplementary Information

Telomere maintenance during anterior regeneration and aging in the freshwater annelid *Aeolosoma viride*

Chi-Fan Chen[1], Tzu-Ling Sung[2], Liuh-Yow Chen[2*], & Jiun-Hong Chen[1,*]

[1]: Department of Life Science, National Taiwan University, Taipei, Taiwan

[2]: Institute of Molecular Biology, Academia Sinica, Taipei, Taiwan

[*]: Corresponding author

Correspondence and requests for materials should be addressed to L.Y.C (email: lyowchen@gate.sinica.edu.tw) & J.H.C (email: chenjh@ntu.edu.tw)

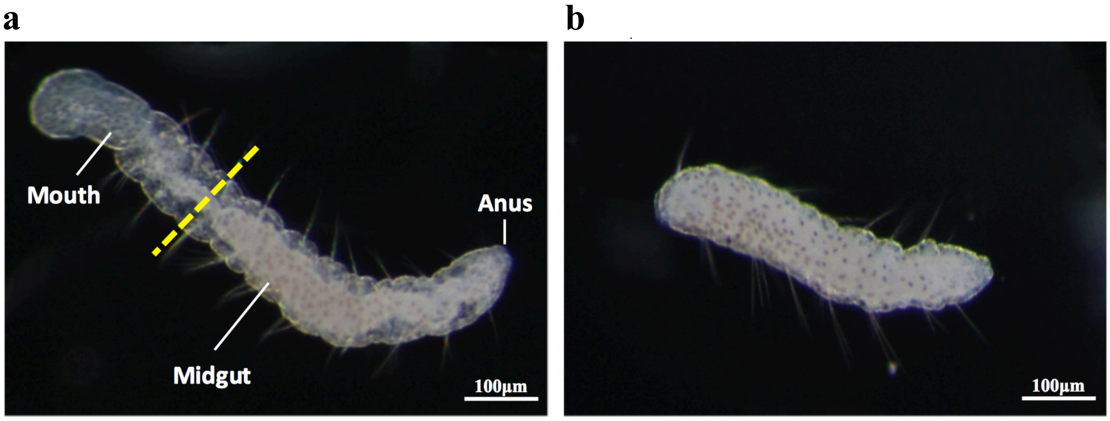

Supplementary Figure 1. Animal manipulation for anterior regeneration. (**a**) Image of a synchronized *A. viride* after 3 days recovery. A new anus has been formed, and the animal is ready to undergo anterior regeneration experiments. To induce anterior regeneration, the animal was bisected at the segment immediately before the expanded midgut. The yellow dashed line indicates the amputation site. (**b**) After amputation, the head region was discarded and the body was transferred to fresh ASW.

CTATGTTAGAGGGCGGTAAGAGTCATTTATTATTTAATGGCACTAAATGATGTTTAACTTCTCTCTGCTACGACAAAAAGAAGAGTTAGTTATTATGGAT

ORF

GTGGAATGACTATACTTGGAAACGTTCATAATAATCAGCAATAATGCTCAATGTTAAATCTATACTGCATCATCTTTTTAAAGAAGTTGTTACTTTGGAA

ORF

GAATTTCTATCAAATTTAATGGGCGCTATGAGGGAAAGTTTAAGAAAATCTGATGATAAAATTTGGTTTAAAAAGTTACTTACTACAACTGTGATTGCCA

ORF

CACCAAAAAAATGGCCGCCCTGCTCCTCAGCATTTGACTTTTTCTGTACAGTCATCACAAAGTGATGTTATTTTAAGAATAATTGAGAGATTAAAAAAAAG

ORF

GGACGAGGTAAATCTGCTATCATTGGGCTACTCTTTGATGTCGGATAATCCAAATGCTCATGTGTCAACATCACCATTTATTGAGAGCACATGTCCAAAT

ORF

CAAAATACTAGTTATCTGCTTACTTTTCCATGGAAAACTCTTCTTGCCAGAATAGGCGACGATGCTATGACGTTTTTACTCGAATGGAGAGTTGTATTCA

ORF

TTGAGGTTCAATCTGGACTTTATCTACAAGTAACTGGTGTGCCTGTATTTGACTTGTGGTCTTGGAAGGTAGGAACAGTGGAAGCAATAAAGGGTTTACC

ORF

GTCAGTATCTCAGGATTTTAATATGCCTTGTGAAATCAATGAGCGAAATATAGACATTGCGGTTGGAAGAAGAAAACGAAAGTTATTTAAACAAATACAA

ORF

AATAGTGATGAAAAAAAACGAGTGTGTAAAAGATTAGAGCAGTATGCCTCCTCTTCTTCTAGCCCTTTGTGTAAAGGAAAGATTGTTCAGTATGCTAATG

ORF

AAACAAATTCAGACATTGGCCATTCTGATATGACTGTTAATTTATTAAAAGAGGCAATGCAAGTAGCCAGCGAAAGAGGTTTCGGTGTTATGTGTTCAAA

ORF

TAGTAGTCAGATTCCCTCTTCTGTTGATTTTAAGAAAGAGGTAATATTGGAAATTAATAATGGAGGTGTGGGTGCAATGAACATCAATGCAAATCAATCT

ORF

GTCAATATTGCTAATATCAAAGAAGCTAATTTTGTAACTGAAAGTCAGTTTAACACAACAAGTGATTCAGGAGTTCCAGCACTGCAAAGATTACCTAAGA

ORF

AAAAAATCATGTCAGTAAGGTCAGGAAGTAATAAAAATTGTGGATTAAGTTCTAATGTGAAAAGAAACAGTGTTGCTCATAAGAAAGGTCAGGTTAAGTG

ORF

CGACATACTCAACAATGTGCATGATAAAATAGTGAAGAAAACTGTTCAAATTCCATGTGCCTGTAATGGTTGCAAATACTGTTGCAGTTATGTTTTCAAG

ORF

AGAAGTAGTATTTTGTACTCGTCTGTGCTTATTGAACGGATGCCAAGCAGTAATTTATTATTCAATCTGGGTGCTGATGTGGTAGGAGCTGAAAAACTTT

ORF

GTACTCATATATTTGCTCACACGCATGAAGGATTCTCAGGTGGTAGTATTTTGCAACACGTGAAATCTTCACAAGTAATTATTTTGTTACAGAATTTGCT

ORF

GTTACAAGTATTAAAAAAACACAAACATTGTCACTATGGCAAGTTATTGGATCATTATTGTTATAACAAAGCCGCTCATTCAGCCACTGAGAAAACTTCA

ORF

AAAAATATTGGCACAGATATGCCAATTTCTCCAAATACTAAGAATTCATCAACTGATGAAAGCAGCTCATTAAACAATTTTTTTCCGCACAGACAGGTAT

ORF

TTTTATTTTTGCGCTTGGCTGTACTAAAAACTATACCATTGGAATTGTTTGGCTCTAAAAAAAAACAGAAATTTATTTTTCAAAAAAATCCAGTTATATGT

ORF

TGGTCTGGGCAAGTTTGAAAAGATGCAGCTCGGTTTTTTTGTCACCAGATTTAAGATAAAGAACACAATATGGACTAAGTTTTTTCATTGCAGTATTTCA

ORF

GCTCAACTAGCTTTTGTTCGTTTTCTTTGTTGGCTTCTTGAAGATGTTATCATGCCATTATTAAAAGGTTATTTCTACATTACTGAAACAATTCAGTATC

ORF

GCAATCGTCTGTTTTATTATCGTAAAAATGACTGGAAATTGGTACGGCAAAAAACTCTAGAAGAATTGACTTTGAAAGGCAATTTGCGGAAAATCTCTGA

ORF

GGTTGATGTAAAGCGACTCCTGAATACTAATAGCACCTTAGGAATTTCTACCATGCGTTTTCTTCCTAAACTTCATGGTTTACGCTGTATATCTAACTTG

ORF

TCAAATTGTAATGCAACTACAGTTACTAAGCAGCAGACATCATCAGTTAACAAGCAGTTGACTCAACTGTTCCAAATACTGACTTATGAAAAGAATAAGC

ORF

ATCTAGAATTAATTGGATCTAGCAAGTTTGGACTTGGTGATATTTATATGTGCTGGAAAAAGTTTGTTCTGCGGCAAAAAGAAAATAATGATTTCAGATC

```
TCTATTCTTTGTGAAAGTGGATATAAGCTGTTGCTATGACTCTATTTTGCAATCAAAGCTGTTTGATATCATAAAGGAAACTCTTGACAAGAGTGATACA
ATGAAGTATGTTATTCGTCGATTTGCCACAGTTAAGTTGCATGGACGCATCCTCAGACGTTCATTTCATCGTGATGCTACTTCAGACGTAGAATTTCAAG
CTAATATGATACTTTATATGAAAGCATTGTCTGCTGCTAAACGACTAAGAAATTGCATTGTTGTTGACCAAGTTGTGCATTCTATAGTGAGGACATCAGA
CCTTCTTAAAAAATTGCGCTTTTTTCTATATCGCAGTATTGTTCGTATAGGACGCAAATACTTTCTGCAACAATTGGGTATTAGTCAAGGAGCAATCATA
TCAACCCTATTATGTAGTTATTATTATGGACACATGGAAGCCAAGTATATACCACATCATCCAGATGATTTGTTGATGAGGCAGGTGGATGACTTTTTGT
TTGTAAGTCCATCAGCTGAACGAGCTATGGATTTTTGTAAAATCATGTCATCAGGAATACCAGAATATAACTGTTACATCAAAAAAGAAAAATGCCTCAC
AAATTTCCCTACTCACAACACAGGAATATCGTTCATTCATGATAATGAATTATTTCCATGGTGTGGAATTTTATTTGACCCTGTGAGTTTAGATGTTTGC
GTAGACTATACCAGATATGCAGGACAAAGTATAGCAGACACACTGTCATTTGACTATGGTTTAGCTCCCGGATTGGTTTTAAAGAGCAAGCTTTTTGAGT
CAATAAGAAGTCGATGCAGTGGCCTATTCTTTGATTCCTTGATAAACAGCAAGAAAGTGATAATCGAGAATCTATTCAAGTTATTCTTATTAACAGCATT
TAGATTTCACACATTAGTCAGGCGTCTACCTAACACACACAGAACAGAATCCAATCCACAGTTCTTTTTAGATATCATCTTAAAATTACCTTTAATTGTT
GAAGAACACTTGAAAACTACAGAAATCAAGTGTGATATTGATGCCACGATAGTCATCCTCATTCCATGGATTTGTTATAAAGCATTTGATTTGAAGTTGA
CTAGACATCATGGCATGTATCAAACAATCCTGAAAATGATAGATCAAATAATACAAAAAACAGAAAACTTACTGAAGTCTGATCTTAGTGATTTTTCTAC
CATCAGTCAGATGCCAACGGAATTCTTGAACATTTTAGACTAAATTTATTGTGAAGATTATGAAACTAACAAATAACTTTCACAATTTACTGTTTTTACA
CAATGAAAACATAATAAAAATTTAAAATGTTTCTGTTTTGCTTTTATCCAAATCTCCATAAGAGCAGAGTATTGTCAAAATTTATTATATTTTTAAAAAT
AGCACATTGAGTTACAGTTTTCACATGGAGTGTGTTATCACAAGGAGTTAGTGTATAATCTTTACAGTGACTGTGCTGTTACGCCAATATAGAATGATAA
ACAAGTCGAGTAAAGTCTGTATAACAGCAGAAACATTTAATCGTCGATTGTAGCAAAGATGAAACTGGAGAATAATACCTAACCAGCTAGGACTCCGGAT
TGTCCCATTACAGTTGAGATAAAACCGCAGAAGCATTACGTAGTCCATTATAGTGAAGATGAAACTGCATGAACATTAGGTAGTTCATTATAATAAACTG
CAGAAACATTAGCTAGTTCATTACAGTGAAGATGAAACAGCAAGAATATTTTATAGTCCATTGCAATAATGATGAAACTGCAGAGACATTTTGTAGTCCA
TTACAGAAAATATAGCATGGTCACTCTCAAAATTAAGAACACGAAAGTTAAAATAGACAGTTCCTAGAAGAGTGATAACCACGCATCAGATGTTATTTTC
GAAAAGCAATAACCACACATCAAATGTTATCTTCGAAGAGCAATTGCCTTGTATTTTCATTTGGTTAGGTTGAGTTGGAGCACAGGAACTATAAACATCT
AATGACAGTCAGTGTGTGTTATGTTGATATGAACTTAAAATAAACCTGAGTAGTTACAAATCTTCATCAAATGCTGACATTGAGTAGTTACAAATCCTGG
TTGAATGCTGACATAAAGCATCACAAAAAATGCTCTAGTATTAGGTCACGTGTTGTTGCCAACATAAGCTATCAGATACTGATGTAACATTTCAAGTCCT
TGCTTTCCCCCATACCTGGGTAGTTTTAAATGCCTAAAATTAGCCTTCAATGCAGCCTTTGATGACCATGTTTCATGAGACAGTGAAGTGACCTATCTTG
ACACTCAACATTAATACTTATAAACACTTACAAACTAACATATTTTGTATTACACAGTGGAAACTTTGGCAATATTATACACTAAAAAAGTTGAGATTTG
TTCTCACATAGTGACCCTAACAAGTTTAGTAATGAACATCCCAATGCATTATTAGCAAAACAAACGTACATGATTGAAATGTACAAAATTAATTAGATTT
ATTAATGCTAATTATTGAAGCATAAATGTATACTTTATGTAAAACACATAAACATATACAGGCGTGCACCACAACTTATACAATCTACAAATAGCAGCAG
AGTTGGTAATAATATAGCACTTGCTTAGCATTTTAGAATAATATCACTCTTGTAATATATATACATGATGCATACTAAAACCACTTATGTATGAACATTA
CACCTGTAAGACAGCAAACATTTTCTACATTCAATTAGATACTTCAATTGAATAACATCATAAGAAGCTAGCTAAATCAAATGAAGTTTTTTTTAAACA
ATATGAATAAAAATACAACTAATAATTTAATATATTAAAAAGAGGGCAATCTGGATAAACATAACCGAATGCAGCACAAAGTAAACTAG
```

Supplementary Figure 2. The *Avi-tert* cDNA sequence was identified using 5' and 3' RACE. Total length is 5389 nucleotides, and the ORF spans residue 144 to 3743 (indicated by the yellow arrow).

```
                                            20                    40                    60                    80                   100
MLNVKSILHHLFKEVVTLEEFLSNLMGAMRESLRKSDDKIWFKKLLTTTVIATPKNGRPAPQHLTFSVQSSQSDVILRIIERLKKRDEVNLLSLGYSLMS
                   120                   140                   160                   180                   200
DNPNAHVSTSPFIESTCPNQNTSYLLTFPWKTLLARIGDDAMTFLLEWRVVFIEVQSGLYLQVTGVPVFDLWSWKVGTVEAIKGLPSVSQDFNMPCEINE
                   220                   240                   260                   280                   300
RNIDIAVGRRKRKLFKQIQNSDEKKRVCKRLEQYASSSSSPLCKGKIVQYANETNSDIGHSDMTVNLLKEAMQVASERGFGVMCSNSSQIPSSVDFKKEV
                   320                   340                   360                   380                   400
ILEINNGGVGAMNINANQSVNIANIKEANFVTESQFNTTSDSGVPALQRLPKKKIMSVRSGSNKNCGLSSNVKRNSVAHKKGQVKCDILNNVHDKIVKKT
                   420                   440                   460                   480                   500
VQIPCACNGCKYCCSYVFKRSSILYSSVLIERMPSSNLLFNLGADVVGAEKLCTHIFAHTHEGFSGGSILQHVKSSQVIILLQNLLLQVLKKHKHCHYGK
                   520                   540                   560                   580                   600
LLDHYCYNKAAHSATEKTSKNIGTDMPISPNTKNSSTDESSSLNNFFPHRQVFLFLRLAVLKTIPLELFGSKKNRNLFFKKIQLYVGLGKFEKMQLGFFV
                   620                   640                   660                   680                   700
TRFKIKNTIWTKFFHCSISAQLAFVRFLCWLLEDVIMPLLKGYFYITETIQYRNRLFYYRKNDWKLVRQKTLEELTLKGNLRKISEVDVKRLLNTNSTLG
                   720                   740                   760                   780                   800
ISTMRFLPKLHGLRCISNLSNCNATTVTKQQTSSVNKQLTQLFQILTYEKNKHLELIGSSKFGLGDIYMCWKKFVLRQKENNDFRSLFFVKVDISCCYDS
                   820                   840                   860                   880                   900
ILQSKLFDIIKETLDKSDTMKYVIRRFATVKLHGRILRRSFHRDATSDVEFQANMILYMKALSAAKRLRNCIVVDQVVHSIVRTSDLLKKLRFFLYRSIV
                   920                   940                   960                   980                 1,000
RIGRKYFLQQLGISQGAIISTLLCSYYYGHMEAKYIPHHPDDLLMRQVDDFLFVSPSAERAMDFCKIMSSGIPEYNCYIKKEKCLTNFPTHNTGISFIHD
                 1,020                 1,040                 1,060                 1,080                 1,100
NELFPWCGILFDPVSLDVCVDYTRYAGQSIADTLSFDYGLAPGLVLKSKLFESIRSRCSGLFFDSLINSKKVIIENLFKLFLLTAFRFHTLVRRLPNTHR
                 1,120                 1,140                 1,160                 1,180                 1,200
TESNPQFFLDIILKLPLIVEEHLKTTEIKCDIDATIVILIPWICYKAFDLKLTRHHGMYQTILKMIDQIIQKTENLLKSDLSDFSTISQMPTEFLNILD*
```

Supplementary Figure 3. The protein sequence of *Avi-tert* translated from its ORF.

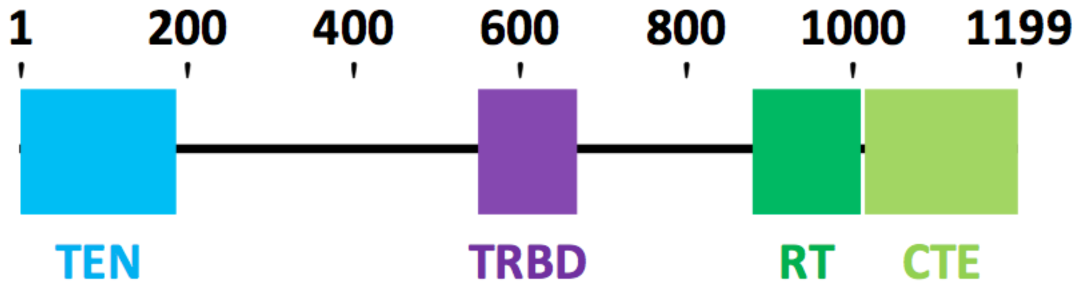

Supplementary Figure 4. Domain annotation of *Avi*-TERT. TEN: Telomerase essential N-terminal domain; TRBD: Telomerase RNA binding domain; RT: Reverse transcriptase domain; CTE: C-terminal extension domain.
