# Supplementary figures and images for: Telomere maintenance during anterior regeneration and aging in the freshwater annelid Aeolosoma viride
Source: Sci Rep. 2018 Dec 24;8:18078. doi: 10.1038/s41598-018-36396-y (PMC6305377; doi:10.1038/s41598-018-36396-y)

Fig. 2a, left

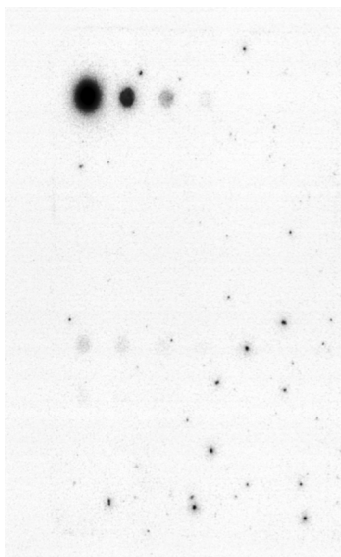

Fig. 2a, right

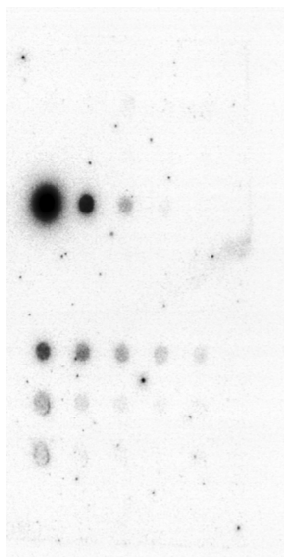

Fig. 2b

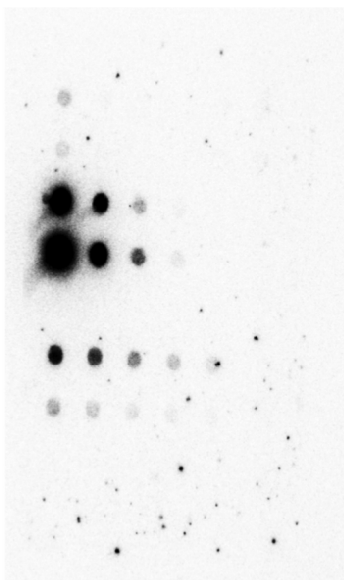

Fig. 2c

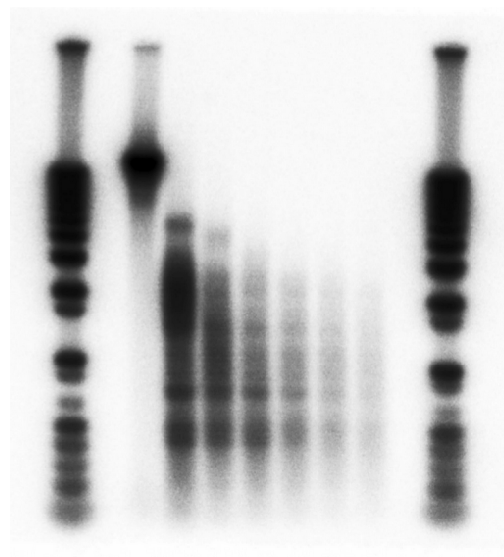

Fig. 3a

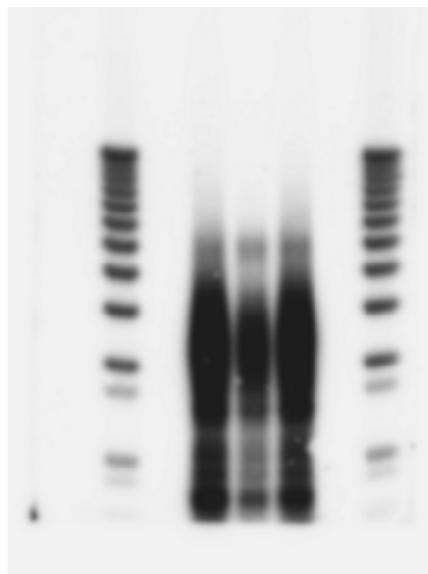

Fig. 3b

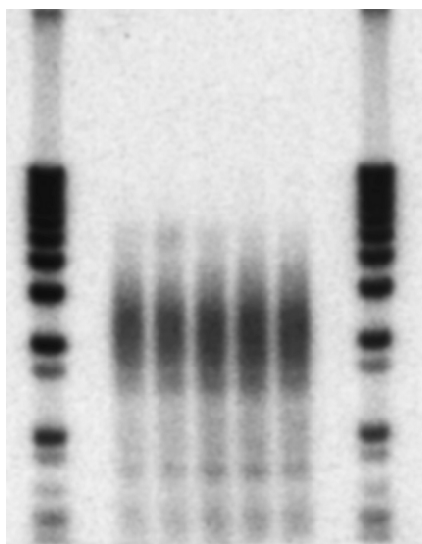

Fig. 4a

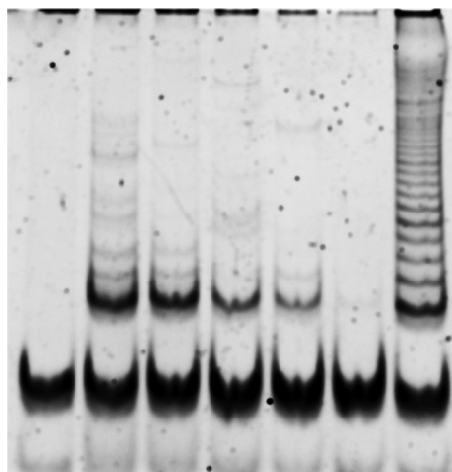

Fig. 4b

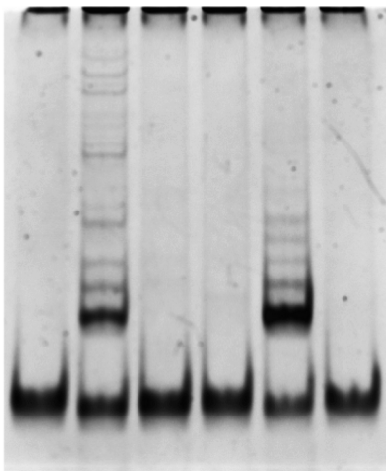

Fig. 7a

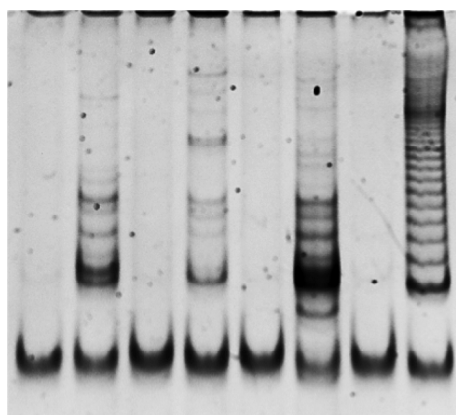

Fig. 7b

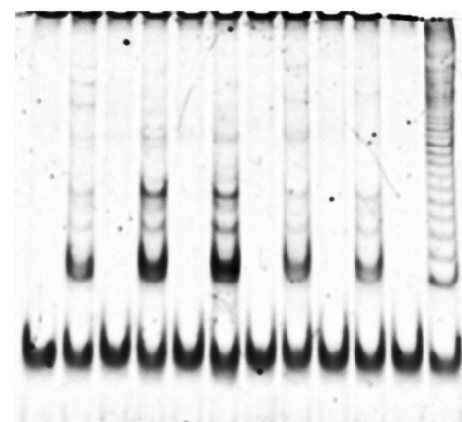

Fig. 8a

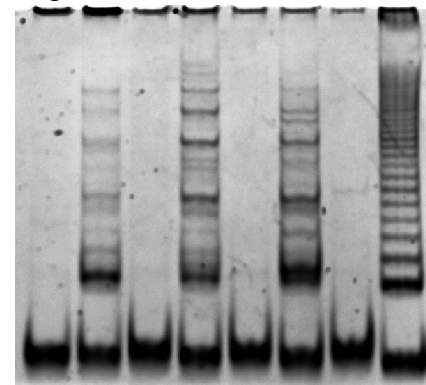

Supplement: Supplementary file 2 — Dataset 1 [file 41598_2018_36396_MOESM2_ESM.pdf]
